# Relationship of Hearing Loss and Tympanic Membrane Perforation Characteristics in Chronic Suppurative Otitis Media Patients

**DOI:** 10.7759/cureus.32496

**Published:** 2022-12-13

**Authors:** Nawal Khurshid, Saleh Khurshied, Muhammad A Khizer, Altaf Hussain, Iqra Safoor, Abdullah Jamal

**Affiliations:** 1 Otolaryngology - Head and Neck Surgery, Pakistan Institute of Medical Sciences, Islamabad, PAK; 2 Otolaryngology - Head and Neck Surgery, Shaheed Zulfiqar Ali Bhutto Medical University, Islamabad, PAK; 3 Ophthalmology, Armed Forces Institute of Ophthalmology, Rawalpindi, PAK; 4 Ophthalmology, National University of Medical Sciences, Rawalpindi, PAK; 5 Otolaryngology - Head and Neck Surgery, Combined Military Hospital, Rawalpindi, PAK; 6 Internal Medicine, United Christian Hospital, Lahore, PAK

**Keywords:** sensory neural hearing loss (snhl), tympanic membrane, chronic suppurative otitis media, otitis media complication, tympanic membrane perforation, conductive hearing loss, hearing loss, csom

## Abstract

Objective

The objective of this study is to assess the relationship between the site of tympanic membrane (TM) perforation and the type and degree of hearing impairment. The secondary objective was to compare the duration of the disease and the degree of hearing loss.

Study design

This is a prospective observational study.

Place and duration of the study

This study was carried out in the Department of Otolaryngology/Head and Neck Surgery at the Pakistan Institute of Medical Sciences, Islamabad, from May 2021 to April 2022.

Patients and methods

Of all the screened patients, 77 fulfilled the inclusion criteria. Patients aged 10-40 years with inactive mucosal chronic otitis media and unilateral perforation in one quadrant were included. The site of TM rupture was observed, and audiometric analysis was performed.

Results

The mean age of participants was 25 ± 8.61 years, with a preponderance of the female gender (57.1%). A total of 32 (41.6%), 19 (24.7%), 19 (24.7%), and seven (9.1%) perforations involved posterosuperior, anterosuperior, anteroinferior, and posteroinferior quadrants respectively. Conductive, mixed, and sensorineural hearing loss was found in 52 (67.5%), 18 (23.4%), and seven (9.1%) cases, respectively. Of all the subjects, 13 (16.9%) had the disease for < one year, 39 (50.6%) for one to five years, 17 (22.1%) for five to 10 years, and eight (10.4%) for > 10 years. There was a statistically significant association between the degree of hearing loss and the site of perforation. No significant association was found between the site of perforation and the type of hearing loss. Duration of disease and degree of hearing loss also had no significant association.

Conclusion

The extent of hearing loss was found to be directly influenced by the anatomical site of perforation, with the posterosuperior quadrant perforation producing the greatest degree of impairment.

## Introduction

Chronic suppurative otitis media (CSOM) is one of the most frequently encountered diseases in ENT practice. It primarily affects children and has remained one of the commonest infectious diseases in the pediatric age group worldwide [1]. The situation is particularly worse in children with poor socioeconomic backgrounds due to inadequate parental knowledge and treatment [2]. CSOM has also been deemed the leading cause of antibiotic prescription in primary healthcare setups [3]. The most typical therapy for CSOM is antibiotics, which kill or inhibit the growth of microorganisms [4]. A significant majority of CSOM cases may require surgical treatment, which involves sealing the rupture in the TM and, if necessary, restoring the ossicular chain [5]. By definition, CSOM is otorrhea of over two to six weeks duration with tympanic membrane (TM) perforation [6]. When the TM is intact, it acts to transfer vibrations from its entire surface area to the footplate of stapes through a chain of ossicles within the middle ear. The TM also protects and acts as a shield to stop the infection from entering the middle ear and maintains a phase difference in sound conduction [7]. Perforation causes a decrease in ossicular coupling due to the inability to transmit pressure across the TM. A study set in the sub-Saharan African region showed a link between hearing loss and TM perforation, including the site of perforation [8]. There is also said to be a strong correlation between age and hearing function in such individuals, especially with conductive hearing loss, which was less severe in children than in patients over the age of 30 [9].

Different studies have shown that CSOM is likely to cause at least a mild to moderate degree of hearing loss in the majority of cases due to its direct effect on air conduction. Factors that affect the degree and type of hearing loss include size, site, and perhaps morphology of TM perforation. In addition, the involvement of the middle ear and its ossicles can independently affect the sound conduction mechanism [10]. Recurrent ear infections in CSOM due to TM perforation cause an increased incidence in the absorption of toxins and macromolecules into the cochlea, which may lead to sensorineural hearing loss (SNHL). Positive correlations have been found between the severity and duration of the disease [11]. Moreover, the frequency of the pathology was found to increase with increasing duration of exposure [12].

A correlation between the site of perforation, type of hearing loss, duration of disease, and degree of hearing loss would help otorhinolaryngologists to adequately determine the treatment of choice. In addition, it should help in a better management plan, thus minimizing the complications of CSOM and providing patients with a better quality of life.

## Materials and methods

The Department of Otorhinolaryngology and Head/Neck Surgery of Pakistan Institute of Medical Sciences (PIMS) Islamabad, a tertiary health care hospital, was the designated site for this study. The institute is categorized as a tertiary care hospital in Islamabad, Pakistan. The institute-associated ethical clearance committee granted formal permission to conduct the study (Ref no.: F.1-1/2015/ERB/SZABMU/539). This prospective observational study was conducted over the course of a year, from May 2021 to April 2022.

The study sample size consisted of 324 patients. Inclusion criteria dictated that patients aged 10 to 40 years were clinically identified as having a quiescent mucosal type of chronic otitis media in the unilateral ear while simultaneously having unimpaired hearing and clinical normalcy in the opposite ear. The research excluded patients with bilateral CSOM, multiple perforations, and a large perforation involving more than one quadrant. Exclusion rendered the patient population to a total of 77. Patients with age-induced hearing loss, accidental damage, and those on previous ototoxic regimens were ruled out for audiometric analysis since hearing loss in these cases was unrelated to CSOM. The criteria were set to include patients who would be compliant in the examination process and, at the same time, exclude those in whom comorbidities may have an independent effect on hearing loss.

A thorough history and demographic data were obtained using a predesigned proforma. A full-size otoscope and a microscope-assisted ear exam were performed on each patient. The orientation of the handle of the malleus was used to identify the side of the ear and the site of the TM perforation. The rest of the anatomical features was also thoroughly examined.

An audiometer, Primus Pro (Auditdata Co, Copenhagen, Denmark), calibrated in accordance with ISO standards, was used in a buffered room, where the audiometric analysis was carried out on patients. Reference frequencies chosen for this study were 250-8000 Hz. When applicable, masking methods were used to estimate the air and bone conduction threshold. Air conduction limits at 500, 1000, 2000, and 4000 Hz were reflective of the hearing level, and the mean calculation of these frequencies was used to assess the hearing. The air-bone gap (ABG) was also measured. An ABG ≥ 20 dB was interpreted as conductive hearing loss, whereas ≤20 dB meant sensorineural hearing loss. The mixed hearing loss meant an ABG ≥ 20 dB, along with a noticeable impairment of bone conduction. WHO grading system [13] was used to characterize hearing loss whereby the degree of hearing loss is mild (25-40 dB), moderate (41-55 dB), moderately severe (56-70 dB), severe (71-90 dB), and profound (>90 dB).

The data were entered and analyzed statistically using the Statistical Package for Social Services v23 (IBM Corp., Armonk, NY) for Windows. Frequencies, percentages, mean values, and ranges were calculated where required. The Chi-square test was used for comparison; a p-value < 0.05 was considered a statistical significance.

## Results

Only 77 patients out of a total of 324 examined fulfilled the criteria for inclusion in the study. Age of participants ranged from 11 to 38 years. The ages of the patients involved are tabulated in Table 1. The mean age of the participants was 25 ± 8.61 years.

**Table 1:** Age of participants

Age range in years (n = 77)	Number of patients	Percentages (%)
10-20	23	29.9
20-30	27	35.1
30-40	27	35.1

Of the total participants, 33 (42.9%) were male, and 44 (57.1%) were female. As patients with unilateral perforation in a single quadrant were included, 41 (53.2%) patients had a perforation in the right ear, while 36 (46.8%) had a perforation in the left ear.

**Figure 1:**
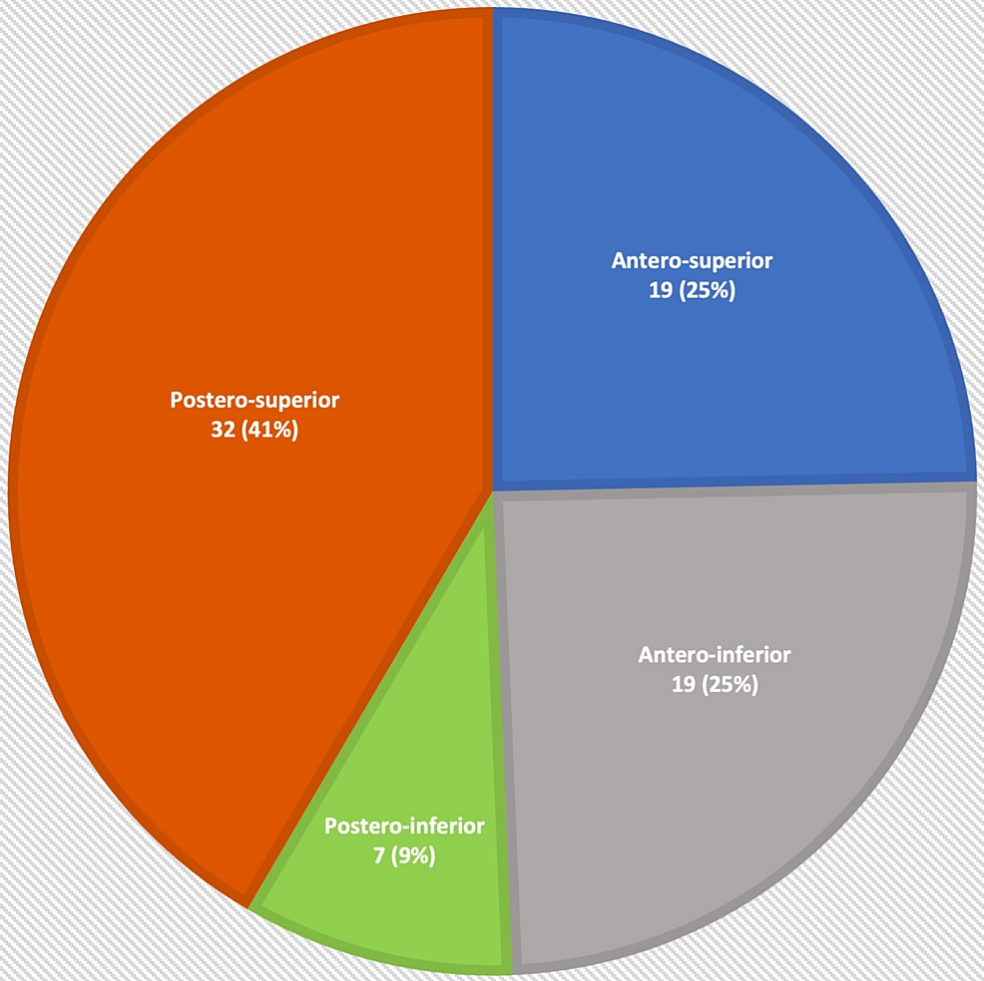
Distribution of patients by anatomical quadrant of perforation

Regarding the site of TM perforation, out of a total of 77 patients included in the study, the posterosuperior quadrant was the most frequently involved at a frequency of 32 (41.6%). Anterosuperior and anteroinferior quadrants had 19 (24.7%) perforations each, while posteroinferior perforation was found in seven (9.1%) cases. Figure 1 is a visual extrapolation of the distribution with respect to anatomical quadrants.

The conductive type of hearing loss was found in 52 (67.5%) of the cases, sensorineural hearing loss was found in seven (9.1%), and mixed hearing loss was found in 18 (23.4%) of cases. In 13 (16.9%) patients, the disease was less than a year old; in 39 (50.6%) cases, the disease was between one and five years old. The disease was between five and 10 years old in 17 (22.1%) cases and more than 10 years old in eight (10.4%) cases.

Different characteristics of TM perforation were also compared with the degree and type of hearing loss. Out of a total of 19 (24.67%) patients with anterosuperior perforation, six (31.6%) had mild, moderate, and moderately severe hearing loss each, while one (5.2%) had profound hearing loss. Similarly, out of a total of 19 patients with anteroinferior perforation, four (21.1%) had mild, 14 (73.7%) had moderate, and one (5.2%) had severe degree of hearing loss. Patients found to have posterosuperior perforation were 32 (41.55%) in number, with 10 (31.2%) having mild, 13 (40.6%) having moderate, four (12.5%) each having moderately severe and severe, and one (3.2%) having a profound degree of hearing loss. Patients with posterosuperior perforation were seven (9.09%) in number, with four (57.2%) having mild, two (28.6%) having moderate, and one (14.3%) having a profound degree of hearing loss. This is presented in tabulated form in Table 2.

**Table 2:** Association of anatomical site of rupture and extent of hearing impairment

Site of perforation	Mild hearing loss n (%)	Moderate hearing loss n (%)	Moderately severe hearing loss n (%)	Severe hearing loss n (%)	Profound hearing loss n (%)	Total n (%)
Anterosuperior	6 (31.6%)	6 (31.6%)	6 (31.6%)	0 (0%)	1 (5.2%)	19 (24.67%)
Anteroinferior	4 (21.1%)	14 (73.7%)	0 (0%)	1 (5.2%)	0 (0%)	19 (24.67%)
Posterosuperior	10 (31.2%)	13 (40.6%)	4 (12.5%)	4 (12.5%)	1 (3.2%)	32 (41.55%)
Posteroinferior	4 (57.2%)	2 (28.6%)	0 (0%)	0 (0%)	1 (14.3%)	7 (9.09%)
Total	24 (31.2%)	35 (45.5%)	10 (12.9%)	5 (6.5%)	3 (3.9%)	77

When the site of perforation was compared with the degree of hearing loss, it was found that there was a statistically significant association between the two, with the posterosuperior quadrant perforation producing the greatest degree of impairment (p = 0.042).

Type of hearing loss was also compared with the site of perforation, and it was found that 11 (57.9%) anterosuperior perforations had conductive hearing loss (CHL), three (15.8%) had SNHL, and five (26.3%) had mixed hearing loss. Regarding anteroinferior perforation, it was found that 13 (68.4%) had CHL, two (10.5%) had SNHL, and four (21.1%) had mixed hearing loss. Patients with posterosuperior perforation had CHL in 22 (68.7%) patients, two (6.2%) had SNHL, and eight (25.1%) had mixed types of hearing loss. Posteroinferior perforations had six (85.7%) cases with CHL and one (14.3%) with mixed hearing loss. Details of these findings are summarized in Table 3. There was no statistically significant association between the two variables (p = 0.820).

**Table 3:** Correlation of anatomical site of rupture and type of hearing loss

Site of perforation	Conductive hearing loss n (%)	Sensorineural hearing loss n (%)	Mixed hearing loss n (%)	Total n (%)
Anterosuperior	11 (57.9%)	3 (15.8%)	5 (26.3%)	19 (24.67%)
Anteroinferior	13 (68.4%)	2 (10.5%)	4 (21.1%)	19 (24.67%)
Posterosuperior	22 (68.7%)	2 (6.2%)	8 (25.1%)	32 (41.55%)
Postreoinferior	6 (85.7%)	0 (0%)	1 (14.3%)	7 (9.09%)
Total	52 (67.5%)	7 (9.1%)	18 (23.4%)	77

When the disease duration was correlated with the degree of hearing loss, no statistically significant association was found between the two (p = 0.294). The data compiled is presented in Table 4.

**Table 4:** Correlation between the duration of disease and degree of hearing loss

Duration of disease (years)	Mild hearing loss n (%)	Moderate hearing loss n (%)	Moderately severe hearing loss n (%)	Severe hearing loss n (%)	Profound hearing loss n (%)	Total n (%)
<1 year	4 (30.8%)	7 (53.8%)	1 (7.7%)	1 (7.7%)	0 (0%)	13 (16.9%)
1-5 years	8 (20.6%)	18 (46.2%)	7 (17.9%)	4 (10.2%)	2 (5.1%)	39 (50.6%)
5-10 years	6 (35.3%)	9 (52.9%)	1 (5.9%)	0 (0%)	1 (5.9%)	17 (22.1%)
>10 years	6 (75%)	1 (12.5%)	1 (12.5%)	0 (0%)	0 (0%)	8 (10.4%)
Total	24 (31.2%)	35 (45.5%)	10 (13%)	5 (6.5%)	3 (3.9%)	77

## Discussion

This study revisited some old as well as a few new aspects in relation to hearing loss associated with TM perforation. Ediale et al. [14] included participants of a younger age group similar to our study. Most of their patients had CHL like ours, and there was a positive relationship between the anatomical location of rupture and the extent of hearing impairment, which was in agreement with our study. Likewise, in studies by Sood et al. [7] and Bhusal et al. [15], the majority of the subjects were of the adolescent age group, similar to our study. Shrikrishna et al. [16] in their study found that the majority of TM ruptures were on the left side, i.e., 53.3%, whereas in our study, the opposite was true with right-sided ruptures at 53.2%. Ibekwe et al. [17] found 41.9% of total perforations in the left ear in their study population.

In this study, we followed a similar pattern as Aslıer et al. [18], who excluded patients with a middle ear pathology in their study to decrease the confounding effect of middle ear pathology on hearing loss. Rajput et al. [19] in their study described that 67.10% of their study population had CHL, 11.80% had mixed, and 16.10% had SNHL, while in our study, the percentages were 67.5%, 23.4%, and 9.1%, respectively; therefore, CHL was found to be the most common type of hearing loss in both studies. Islam et al. [20] found that 80.8%, 17.17%, and 2.01% had CHL, mixed hearing loss, and SNHL, respectively, which is also in agreement with the trend in our study. Kumar et al. [21] reported in their study that their study population had CHL in the vast majority of cases (>90%), while in our study, although the most common type was still CHL, it was overall less in number (67.5%). CHL was the main type of hearing loss in all the above studies, including ours. The only study having published a significantly higher amount of SNHL patients was by Rajput et al. [22], which showed SNHL to be present in 19.5% of their CSOM study population. This difference was likely due to the inclusion of patients with middle ear pathology, the same group that was excluded from our study.

Maharjan et al. [23] and Nepal et al. [24] demonstrated a statistically significant correlation, which was in agreement with our data, between the location of the perforation and hearing loss. In contrast, a conflicting view was presented by Mehta et al. [25], stating that the effects of the location of TM perforation and hearing, if any, were small and insignificant.

Castelhano et al. [26] established that conductive hearing loss as a consequence of TM rupture with higher losses was seen when involved by inflammatory backgrounds, larger perforations, the posterior quadrant involvement, or involvement of manubrium, which was in agreement with our study. Balci et al. [27] correlated the position of TM perforation to the severity of hearing loss, emphasizing the shape of the perforation. Recording of perforation shape was not part of our methodology. Still, it may have significance based on the fact that greater hearing loss was associated with posteroinferior perforation in their study, which was in contrast to our results. Studies by Kim et al. [28], Casale et al. [29], and Rana et al. [30] had a partially similar deduction as Balci et al. [27], in which the association between the morphological aspects as well as the size of the perforation was said to be a separate factor affecting the severity of hearing loss independent of the site of perforation.

Regarding a link between the severity of hearing loss and the duration of the TM perforation, relatively less published literature is present. Pannu et al. [10] in their study had 28% of patients with a disease duration of less than one year, 35% had the disease for one to five years, and 37% had the disease for more than five years, while the findings in our study were 16.9%, 50.6%, and 33.1%, respectively. Maharjan et al. [23] found a major connection between the period of otologic disease and hearing loss. In contrast, Sood et al. [7] refuted any connection between the two, which was similar to our study results. The varying results in this aspect could perhaps be attributed to the fact that the duration of the disease was noted based on patients' memory and perception of the disease, which can vastly differ between individuals and is liable to patients correctly remembering the duration.

Another aspect of CSOM that deserves mention is its negative effect on the quality of life (QoL). Various studies have shown a statistically significant improvement in hearing threshold and overall QoL in vastly different patient groups (children, adults, swimmers, etc.) who had undergone corrective surgery, especially tympanoplasty, for TM perforation [31-35].

Limitations of our study include relying on patients' history for recording the duration of disease, relying on a single examination to confirm a quiescent type of disease, and relying on a clinical examination instead of photographic evidence by the use of a video otoscope, which is always preferable for future reference and confirmation of signs.

Strengths of this study include a relatively large sample size based on the inclusion and exclusion criteria and assessment of the association between many different aspects of hearing loss associated with tympanic membrane perforation, which has usually been done separately in the literature, that too on different population groups with little connection to each other.

## Conclusions

Our research has led us to conclude that the location of the perforation affects the extent of hearing impairment experienced by the patient, with the perforation in the posterior superior quadrant causing the most significant impairment. In addition, conductive hearing loss was the most frequent type of hearing loss encountered in these cases. However, it was also noted that the site of perforation plays no significant role in determining the type of hearing loss, with most subjects having mild to moderate hearing loss. In addition, when hearing loss and disease duration were compared, it was discovered that an extended course of illness had no discernible impact on the severity of hearing loss.
